# Application of Genetic, Genomic and Biological Pathways in Improvement of Swine Feed Efficiency

**DOI:** 10.3389/fgene.2022.903733

**Published:** 2022-06-09

**Authors:** Pourya Davoudi, Duy Ngoc Do, Stefanie M. Colombo, Bruce Rathgeber, Younes Miar

**Affiliations:** Department of Animal Science and Aquaculture, Dalhousie University, Truro, NS, Canada

**Keywords:** pig, feed efficiency, heritability, candidate genes, biological pathways, genetic improvement

## Abstract

Despite the significant improvement of feed efficiency (FE) in pigs over the past decades, feed costs remain a major challenge for producers profitability. Improving FE is a top priority for the global swine industry. A deeper understanding of the biology underlying FE is crucial for making progress in genetic improvement of FE traits. This review comprehensively discusses the topics related to the FE in pigs including: measurements, genetics, genomics, biological pathways and the advanced technologies and methods involved in FE improvement. We first provide an update of heritability for different FE indicators and then characterize the correlations of FE traits with other economically important traits. Moreover, we present the quantitative trait loci (QTL) and possible candidate genes associated with FE in pigs and outline the most important biological pathways related to the FE traits in pigs. Finally, we present possible ways to improve FE in swine including the implementation of genomic selection, new technologies for measuring the FE traits, and the potential use of genome editing and omics technologies.

## 1 Introduction

Since pig producers profit is affected by both inputs and outputs, it is essential to apply methods for reducing input costs as the goals are to improve production and increase profit (Hume et al., 2011). Providing feed for an animal is one of the main input costs in any animal production system. The expenses associated with feed can account for 60–70% of total costs for pig production systems (Patience et al., 2015). Feed efficiency (FE) can be defined as the association between feed intake (input) and production (output), and therefore, improving FE will reduce production costs. Another main goal of improving FE is to reduce the negative impacts of production on the environment. The livestock production sector is a major source of worldwide greenhouse gas (GHG) emissions, notably methane (CH_4_), nitrous oxide (N_2_O) and carbon dioxide (CO_2_). It has been reported to contribute 14.5% of the global anthropogenic GHG emissions (Gerber et al., 2013). Therefore, it is critical to find a strategy to mitigate GHG emissions and reduce the impact of animal agriculture on climate change. Improving FE can reduce the environmental footprint as there is a significant correlation between FE and methane emission traits (Connor et al., 2012). Animals with high FE use energy from feed more efficiently, which ultimately reduces the enteric methane production and GHG emissions (Basarab et al., 2013). A lower protein degradation rate in more efficient animals may lead to reduced environmental pollution from nitrogen, as a result of more efficient nitrogen utilization (Bezerra et al., 2013). Several studies reported improved FE as a promising strategy to reduce GHG emissions, and consequently, reduce global warming (Fitzsimons et al., 2013; Dini et al., 2019; Soleimani and Gilbert, 2021).

In order to relate feed intake to animal efficiency, it is necessary to apply comprehensive strategies for FE measurements. One of the most common measures of FE is feed conversion ratio (FCR) that is defined as the ratio of body weight gain to feed intake (Skinner-Noble and Teeter, 2003). Another measure of FE is residual feed intake (RFI), as proposed by Koch et al. (1963). It is described as the difference between actual feed intake and expected intake based on an animal’s body size and maintenance over a time period.

FE traits are economically important traits that are controlled by many genes and environmental factors. Several studies have been conducted to analyze these traits. Examples include, evaluation of breeding values for selection candidates (Miar et al., 2015; Garrick, 2017) or identifying candidate genes for FE (Do et al., 2014a; Fu et al., 2020). Some studies successfully identified quantitative trait loci (QTLs) that are related to feed efficiency in livestock species (Andersson et al., 1994; Georges et al., 1995), however, due to low resolution in QTL mapping analysis and complex genetic architecture in most QTLs, QTL mapping has not been very successful (Andersson, 2009). Genome-wide association study (GWAS) is a powerful tool for identification of causal genes or regulatory elements for economically important traits such as FE traits in livestock. GWAS has some benefits, e.g. the power to identify genetic variants and the practical approach to evaluate genetic architecture of complex traits (Kronenberg, 2008). Genomic data are extensively accessible in the livestock industry and supply a profitable means of estimating genetic merit. This is helpful for selection decisions that enhance genetic gains. Genomic selection is now a widely used animal breeding program approach since it enhances the selection for difficult and expensive traits to measure, such as FE and growth traits (Meuwissen et al., 2013). Genomic selection can increase genetic gain by reducing generation interval as breeders are able to predict the genetic potential of animals at an early stage of life (Meuwissen et al., 2013; Miar et al., 2015). However, the largest benefit of genomic selection for swine will be from an increase in the amount of genetic gain by improved accuracy of genomic estimated breeding values (GEBVs) compared with traditional approaches that ignore Mendelian sampling (Wiggans et al., 2017). In the current review, we summarize the different measurements of FE, then present the state of the art for genetic and genomic studies for FE in pigs. This is followed by an overview of the critical biological pathways linked to the regulation of FE. Finally, there is a discussion of current and future methods and technologies that could improve FE in pigs.

## 2 Measures of Feed Efficiency and Non-genetic Factors Effects

Body weight gain per unit of feed consumed by an animal is a general measurement of FE in most studies (Patience et al., 2015). Although FE has conventionally been described as the ratio of feed consumed compared to the growth achieved by an animal, other FE indicators have been proposed recently (Crowley et al., 2010; Berry and Crowley, 2012). Despite the importance of FE as a critical parameter in breeding programs, there is little consensus regarding the optimized approach to achieve ideal FE (Gaines et al., 2012). Firstly, this lack of concensus might be due to the fact that a complex biological process affects FE (Cantalapiedra-Hijar et al., 2018). Secondly, there is controversy surroundinht be due to the fact that a complex biological process affects FE (Cantalapiedra-Hijar et al., 2018). Secondly, there is controversy surrounding how to define and measure FE traits (Patience et al., 2015). Finally, it should be noted that measuring FE is remarkably difficult to measure; compared to growth traits, which can easily be obtained by weighing the animals at specific periods in a lifetime (Hoque and Suzuki, 2009).

FE can be defined using energy consumed as a factor instead of the feed consumed by the animal as part of the calculation (Patience, 2012). This approach can be beneficial as energy is a significant contributor to the cost of diet (Patience, 2012). However, the inaccurate estimation of energy concentration in the diet is one of the main drawbacks of this method, as there are issues at the time of quantifying dietary energy concentration (Patience, 2012). In addition, the differences in methods expressing the dietary energy concentration like digestible (DE) and metabolizable (ME) energy might cause differences in FE due to the artifacts of the inaccurate energy system (Patience, 2012).

**FIGURE 1:**
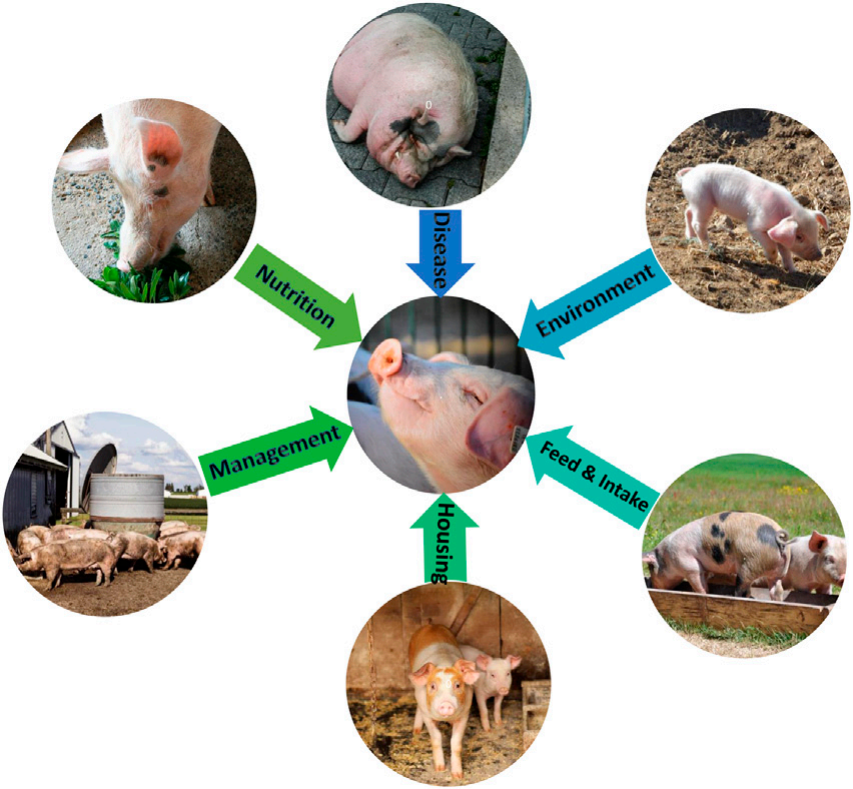
Non-genetic factors affecting feed efficiency traits in swine industry. All pictures were taken from public-domain share-free websites.

Despite the genetic capability of FE traits in swine, there are numerous internal and external factors affecting FE in pigs that impede to capture the full genetic potential of animals. These factors include nutritional elements (the quantity, composition, and the digestibility of feed), maintenance processes, immune function, thermal environment and access to feed and water (Knap and Wang, 2012). Figure 1 illustrates the factors that might affect FE in the swine industry. Studies have shown that FE in pigs can be improved by increasing the concentration of energy in the diet (De la Llata et al., 2001; Beaulieu et al., 2009). However, it is necessary to consider the process of growth, as the energy requirements vary through different growth phases (Kil et al., 2013). Although increasing dietary energy concentration may lead to improved feed efficiency, the correlation between dietary energy concentration and FE is very low (Oresanya et al., 2008). This might be due to non-dietary factors affecting estimation of FE, such as inaccurate measurement of, dietary energy concentration, and animal variation (Patience et al., 2015).

### 2.1 Feed Conversion Ratio

With respect to improving FE in the pig industry, feed conversion ratio (FCR; feed to body weight gain ratio) has traditionally been investigated as a simple and relatively common indicator of FE (Hoque and Suzuki, 2009). Although FCR is considered an essential part of the goal in pig breeding programs, there are some definite issues with FCR. Selection based on FCR can lead to large-sized animals at a mature age that might have high energy requirements for maintenance (Smith et al., 2010). In addition, high genetic correlations with FCR, growth, body size and body composition, cause the changes in component traits in future generations (Gunsett, 1984). Finally, animals with similar FCR might vary in growth rate and feed intake (Smith et al., 2010). Thus, there is a need to define an indicator that negates the effect of body weight on FE of each animal.

### 2.2 Residual Feed Intake

Residual feed intake (RFI) is another FE measurement proposed as an alternative to FCR. RFI is defined as the difference between the observed feed intake and the expected feed intake. This was first proposed by Koch et al. (1963). They suggested that feed intake could be adjusted for body weight and weight gain. Since RFI is independent of body weight and average daily gain (ADG), selection for RFI can alter the energy of maintenance requirements without changing the body size and production level. In addition, due to the RFI’s mathematical independence to animal production, this method is notably suitable to investigate the biological mechanisms underlying the FE variation in each individual (Berry and Crowley, 2013). However, RFI calculation might be dependent on the predicted feed requirement for production and maintenance (Do et al., 2013a), which might cause difficulty in comparing results of different studies. In addition, in the case where genetic correlation exists between FE and maintenance traits, the heritability estimation might be unreliable (Lu et al., 2015).

### 2.3 Other Feed Efficiency Indicators

A wide variety of terms have been proposed to define FE, which can be applied as alternative measurement for FCR and RFI (Berry and Crowley, 2013). Kleiber ratio (KR), defined as growth rate/body mass^0.75^, was suggested as an indirect selection parameter for feed conversion (Kleiber, 1947). It is acknowledged that KR is a useful indicator in selection for growth efficiency since it does not require the calculation of individual intake and enables ranking of individuals with high growth efficiency relative to body size (Köster et al., 2016). Another indicator of FE is partial efficiency of growth (PEG), described as the ratio of ADG per unit of feed intake consumed for growth. Studies reported that PEG has some advantages over FCR since it has considerably lower genetic and phenotypic correlation with ADG compared to ADG and FCR (Arthur et al., 2001; Nkrumah et al., 2004). Residual gain (RG) and residual feed intake and gain (RIG) are other alternative measures of FE (Crowley et al., 2010; Berry and Crowley, 2012). RG is defined as the difference between the actual ADG and the expected ADG and combines the measurements of growth and feed intake in a similar principle to RFI. However, for RFI, feed intake is regressed on ADG and body weight, but in the calculation of RG, ADG is regressed on feed intake and body weight (Crowley et al., 2010). RIG, proposed by Berry and Crowley (2012), combines RFI and RG to identify efficient and fast-growing animals independent of their body weights. Therefore, the advantages of both reduced feed intake and greater ADG are represented in RIG. However, it is necessary to perform comprehensive RIG trait examinations in swine studies to support its advantages to the swine industry (Lu et al., 2017).

## 3 Genetics of Feed Efficiency

### 3.1 Heritability Estimates of Feed Efficiency and Its Components Traits

Generally, estimated heritability of traits (defined as the ratio of genetic variation to the overall phenotypic variation) is used to determine the degree to which traits are under genetic control. In order to have an accurate estimate of heritability, a well-established measure of FE, as well as complete and precise pedigree information on many individuals are required. Supplementary Table S1 provides an overview of literature for heritabilities of FE-related traits in pigs.

Among all FE traits in pigs, RFI has received increasing attention in recent years (Godinho et al., 2018; Nascimento et al., 2019; Santiago et al., 2021). The heritability estimates for RFI have been reported in the range of 0.10–0.51 (Jiao et al., 2014a; Hong et al., 2021). The diverse range of heritability estimates presented in the literature is due to evaluation of different populations, ages, diets, environments, and the number of animals in the study. A controversial subject related to RFI is that this term or other unspecified feed intake terms are referred to as FE. The residual might be associated with random errors, for example, prediction and measurement errors, inaccurate recording or feeding loss (Van Der Werf, 2004). These errors can reflect the phenotypic variation, which might change the heritability of RFI. Several studies have evaluated the heritability of FCR in different pig populations (Supplementary Table S1). The heritability of FCR in pigs has been reported to vary considerably from 0.13 to 0.49 (Habier et al., 2009; Hong et al., 2021). Different studies also estimated the heritability for ADG and daily feed intake (DFI) as other FE component traits (Supplementary Table S1). The average estimates of heritability reported for component traits (such as ADG and DFI) were higher compared to RFI and FCR but still varied substantially, ranged from 0.23 to 0.67 and 0.16 to 0.66 for ADG and DFI, respectively (Supplementary Table S1). Published studies indicated that FE-related traits are moderately heritable traits, and therefore, have good potential to respond to selection (Supplementary Table S1).

### 3.2 Genetic Correlation of Feed Efficiency With Other Traits

**FIGURE 2:**
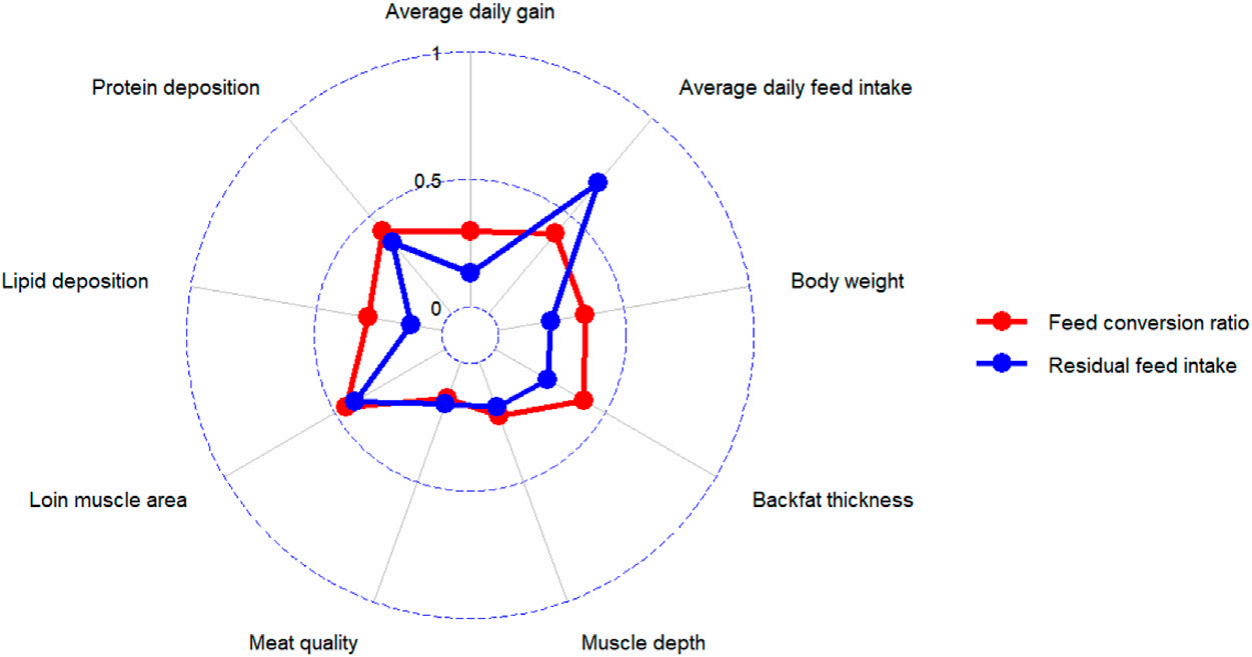
The average absolute values of genetic correlations of RFI and FCR with other economically important traits in pig.

Although heritability can provide information about the candidates’ genetic merit for traits of interest, understanding the magnitude and direction of correlations between FE traits and other economically important traits is essential to establish a successful breeding program (Brito et al., 2020). Traits with high positive genetic correlations tend to improve simultaneously, but high negative genetic correlations between traits cause the opposite direction of improvement. The average absolute values of genetic correlations of RFI and FCR with other economically important traits such as ADG, DFI, body weight, backfat thickness, muscle depth, meat quality, loin muscle area, lipid deposition, and protein deposition were calculated based on the studies published between 1995 and 2022 (Figure 2).

The FCR and RFI genetic improvement strategies are different and rely on their particular genetic correlations with other production traits. Genetic correlations between FCR and RFI traits are high and positive and range from 0.53 to 0.95 (Hoque and Suzuki, 2008; Jiao et al., 2014a). Previous studies indicated that FCR had moderate positive correlations with body weight and ADG (Herrera-Cáceres et al., 2019; Hong et al., 2021); so FCR could be changed indirectly with the selection of growth traits. In contrast, indirect selection of growth traits including body weight, ADG and in some cases, backfat thickness (BF), would not affect RFI, as the calculation of RFI is based on the regression of feed intake on growth traits (Kennedy et al., 1993). In pigs, the genetic and phenotypic correlations of RFI and ADG are relatively low and range between -0.06 and 0.28 (Hoque et al., 2007; Saintilan et al., 2011). The magnitude of genetic correlation between RFI and BF is reported to be low and close to zero in different studies (Saintilan et al., 2011; Godinho et al., 2018; Hong et al., 2021). The moderate negative correlation between RFI and body leanness in different studies (Cai et al., 2008; Hsu et al., 2015) indicated that selection based on RFI might increase lean growth. Selection based on RFI is also associated with animal characteristics related to energy cost. Decreasing the maintenance energy requirements leads to decreased physical activity and reduced heat production of pigs, which could significantly contribute to higher energy efficiency (Gilbert et al., 2017). Therefore, low RFI pigs are desired since they spend less energy on feed consumption, interacting with others, heat production, and maintenance requirements (Gilbert et al., 2017). RFI is reported to be highly correlated with DFI, indicating that selection against RFI can decrease DFI (Von Felde et al., 1996; Gilbert et al., 2007). Although FCR was positively correlated with DFI, the magnitude was less than for RFI and ranged from 0.13 to 0.88 (Von Felde et al., 1996; Do et al., 2013a). Therefore, selection for low RFI would decrease DFI more than FCR-based selection in pigs. Nevertheless, it is important to consider differences in body weight of animals using in genetic parameter estimation, since animals with different weights might have different maintenance requirements, and thereby have an impact on the estimated parameters, genetic correlations and prediction of FE traits (Patience et al., 2015).

## 4 Genomics of Feed Efficiency

### 4.1 QTL and Candidate Genes Associated With Feed Efficiency

Detection of causative mutations underlying QTLs has always been challenging in domestic animals (Zhang et al., 2012). Compared to conventional QTL mapping methods, GWAS has the power to identify genetic variants with even modest effects (Hirschhorn and Daly, 2005). In pigs, the advancement of genomic technologies has enabled researchers to perform different GWAS studies to identify genomic regions and candidate genes associated with economically important traits, including meat quality and quantity, reproductive traits, and FE traits. To date, 34,342 QTLs for 708 different traits have been mapped in the pig genome (Hu G et al., 2022). Taking different FE indicators into account, 410 QTLs for FCR, 96 QTLs for RFI, 143 QTLs for DFI, and 815 QTLs for ADG presented in the animal QTL database by December 2021 (Hu G et al., 2022). The descriptive information of GWAS studies for FE traits in pigs is shown in Supplementary Table S2. In various studies, a large number of regions with small effects were identified for FE traits in pigs, suggesting that feed efficiency is a polygenic trait (Onteru et al., 2013).

Feeding is one of animals’ most conserved activities, and a key mechanism for survival is to regulate feed intake (Bader et al., 2007). FE traits are quantitative traits with complex genetic architecture (Do et al., 2014b). Therefore, an important field of research in livestock genetics and breeding is the discovery of candidate genes underlying these traits. To date, several studies investigated genes that potentially affect FE in pigs. Notably, one of the well-known candidate genes for growth traits or/and feed intake in pigs is the melanocortin-4 receptor (*MC4R*) located at 178 Mb on SSC1 (Kim et al., 2000; Piórkowska et al., 2010; Onteru et al., 2013). The *MC4R* is an integral part of the nervous system and plays an essential role in the regulation of feed intake, energy balance, and body weight in mammals (Adan et al., 2006). In pigs, several GWAS studies showed that the *MC4R* gene could be associated with body weight (Lee et al., 2020), backfat thickness (Wang et al., 2019), ADG (Howard et al., 2015), and ADFI (Onteru et al., 2013).

**FIGURE 3:**
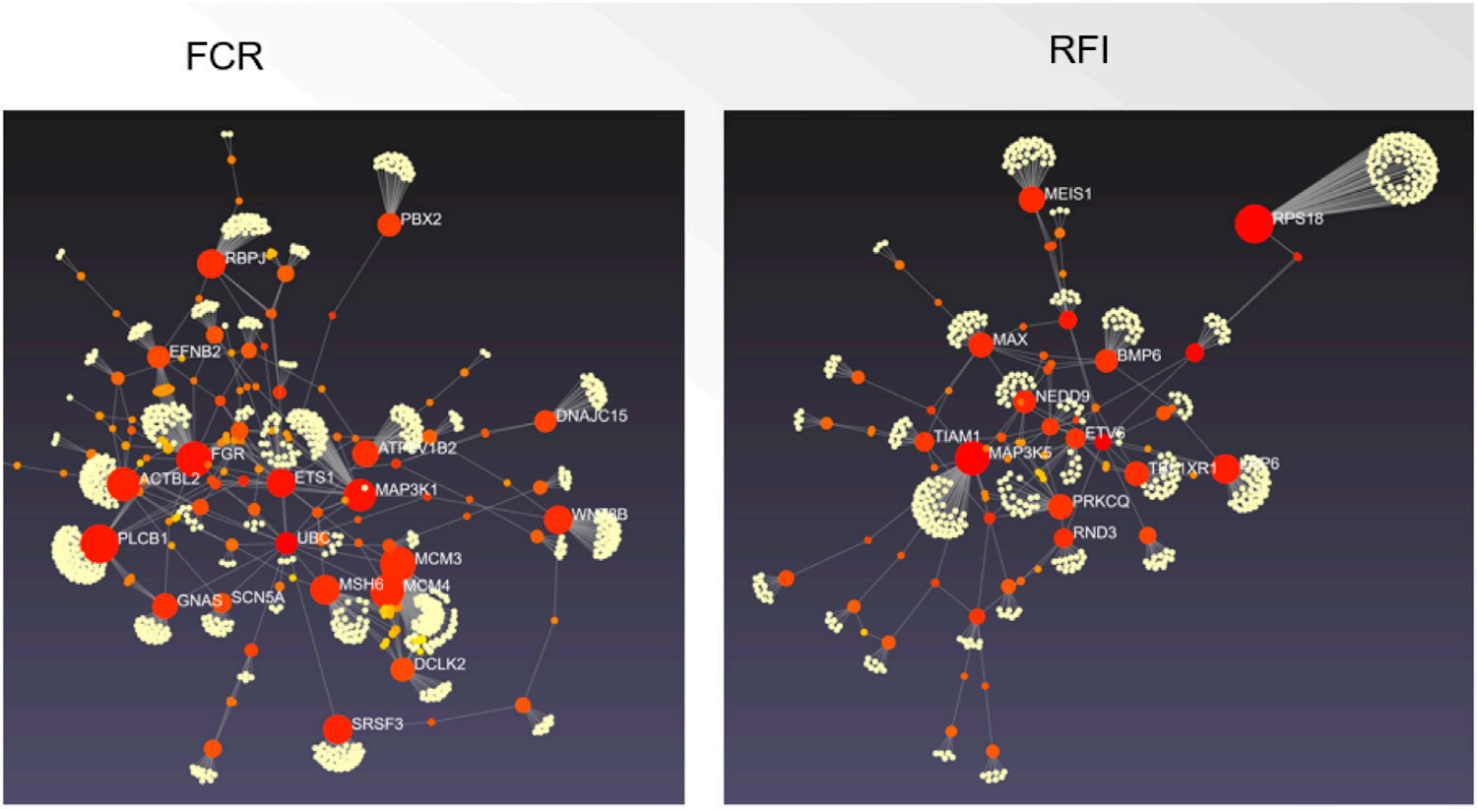
The protein protein interaction networks for candidate genes for feed conversion ratio (left) and residual feed intake (right) in pig. The candidate genes from the pig QTL database and additional recent papers (Supplementary Table S2) were used for building the interaction network using the default inputs of Network Analyst (Zhou et al., 2019).

High-mobility group AT-hook 1 (*HMGA1*) gene is another promising candidate gene, as it is functionally related to several FE-related traits (Guo et al., 2015; Qiao et al., 2015; Ji et al., 2019). Guo et al. (2015) indicated that *HMGA1* was close to the top SNP in the strongest associated region detected on SSC7. They proposed the *HMGA1* gene as a candidate gene for FCR on SSC7. *HMGA1*, which encodes a chromatin-associated protein, is ubiquitous in all cells of higher eukaryotes (Cleynen and Van de Ven, 2008) and is known to have an essential biological role in cell growth and differentiation by acting as a dynamic regulator of chromatin structure (Bustin and Reeves, 1996; Melillo et al., 2001). *HMGA1* affects the expression of insulin growth factor-binding protein (*IGFBP*) and therefore, can serve as a modulator of insulin-like growth factor 1 (*IGF1*) activity and consequently regulates glucose uptake (Iiritano et al., 2012). Several studies have reported that the *IGF1* gene is involved with body size and body height in both humans and animals (Sutter et al., 2007; Okada et al., 2010). Furthermore, Kubota et al. (1999) reported that *HMGA1* could bind with peroxisome proliferator activated receptor gamma (*PPARG*), a crucial regulator of fat-cell differentiation and glucose homeostasis. In pigs, *HMGA1* might have pleiotropic effects on several traits, as *HMGA1* variants have been associated with growth (Guo et al., 2015), fatness (Qiao et al., 2015), and carcass traits (Gong et al., 2019). Ji et al. (2019) reported a suggestive QTL (near the *HMGA1* gene) located on SSC7 (34 Mb) for nine different body weight and ADG traits. The *HMGA1* gene near this region supports the pleiotropic effect of this QTL. Interestingly, Quan et al. (2018) identified another member of high mobility group AT-hook family, *HMGA2*, in a haplotype block located on SSC5 spanning 774 Kb of chromosome-wide significant SNP (ID= H3GA0016186) for ADG. The H3GA0016186 was located 78,423 bp upstream of *HMGA2* on SSC5. Abi Habib et al. (2018) demonstrated that *HMGA2* directly regulated *IGF2* through increased expression of pleomorphic adenoma gene 1 (*PLAG1*); and any defects in the *HMGA2–PLAG1–IGF2* pathway can lead to the Silver–Russell Syndrome, a syndromic form of fetal growth retardation. Further studies on porcine *HMGA2* should be conducted in the future to thoroughly investigate the role of *HMGA2* on FE in pigs. Figure 3 depicts the hub genes based on the protein protein interaction networks for RFI and FCR.

### 4.2 Biological Pathways Related to Feed Efficiency

The high number of candidate genes and localization of QTLs indicate that the genes underlying FE traits in pigs are involved in numerous biological processes. As discussed before, several GWAS studies have been reported some SNPs and candidate genes that might be associated with FE in pigs (Do et al., 2014a; Fu et al., 2020; Miao et al., 2021). However, identifying biological pathways affecting FE is challenging, and GWAS studies are limited in finding the chromosomal regions or preselected genes that might be related to FE (Singer, 2009). Instead, a growing number of studies have applied omics methods to explore the mechanisms affecting FE in pigs, including transcriptomics (Vigors et al., 2019; Xu et al., 2020a), 16S rRNA gene sequencing (Quan et al., 2018; Si et al., 2020), proteomics (Wu et al., 2020), and metabolomics (Carmelo et al., 2020; Wang and Kadarmideen, 2020). By screening and analyzing the differentially expressed genes (DEGs) and related biological pathways derived from transcriptomics studies, candidate genes and pathways affecting FE can be identified (Nagalakshmi et al., 2008; Wilhelm and Landry, 2009). Metabolites are downstream of the gene regulation network in the biological processes from DNA through RNA to proteins; therefore, the metabolite displays more detailed biological terminal information. As the final products of cell metabolism, metabolites are the ultimate response of biological systems related to genetic change; hence, metabolomics is regarded as the link between genotypes and phenotypes (Fiehn, 2002). It is also acknowledged that proteomics approaches can be applied to examine the changes in protein expression of liver and muscle; hence proteome analysis is essential to fully understand the regulatory mechanism of animals (Kuhla and Metges, 2013). Moreover, the main information on the biological process is mainly explored at the protein level, so investigating FE at the protein level will provide information on the mechanism of a trait under different physiological or pathological conditions (Bassols et al., 2014; Wang et al., 2017). To date, several omics studies have been carried out in pigs and have revealed various molecular mechanisms of FE, including carbohydrate and lipid metabolism (Horodyska et al., 2019a), energy metabolism (Xu et al., 2020b), and immune responses (Vigors et al., 2019).

#### 4.2.1 Metabolism-Related Pathways Involve in Feed Efficiency

Metabolic pathways play an essential role in regulating FE in pigs. Studies indicated that lipid metabolism is enriched by down-regulated genes of adipose tissues in high FE pigs (Horodyska et al., 2019b), and up-regulated genes are involved in lipid catabolism (Gondret et al., 2017). Cyclic adenosine monophosphate (cAMP) and Ca^2+^ are the two most common second messengers in eukaryotic cells (Katona et al., 2015) that are closely related to lipid metabolism (Benmansour et al., 1991; Chen et al., 2004; Goudarzi et al., 2018). Xu et al. (2020a) carried out a transcriptomics experiment to detect DEGs of Yorkshire pigs with extremely high and low FE. Their results indicated that DEGs were significantly related to the cAMP signaling pathway and Ca^2+^ binding. The high level of Ca^2+^ exerts an antilipolytic effect by activation of phosphodiesterase (PDE), leading to the inhibition of lipolysis through reducing cAMP and hormone-sensitive lipase (HSL) phosphorylation (Xue et al., 2001). Jing et al. (2015) reported several mRNAs and miRNAs differentially expressed in skeletal muscle of purebred Yorkshire boars with different RFI. The down-regulated genes such as *FABP3*, *RCAN*, *PPARGC1* (*PGC-1*), *HK2* and *PRKAG2* were mainly involved in mitochondrial energy metabolism regulatory pathways, whereas the up-regulated genes, including *IGF2*, *PDE7A*, *CEBPD*, *PIK3R1* and *MYH6* were involved in skeletal muscle differentiation and proliferation. Peroxisome proliferator-activated receptor γ coactivator-1 (*PGC-1*) plays a vital role in mitochondrial biogenesis as it activates cAMP response binding protein and nuclear respiratory factors (Asin-Cayuela and Gustafsson, 2007). Similarly, Fu et al. (2017) reported that mitochondrial energy metabolism in skeletal muscle tissue was significantly associated with FE in purebred Yorkshire pigs. Recently, Carmelo and Kadarmideen (2020) applied multiple types of transcriptomic analysis; and their results supported the knowledge that mitochondrial activity had a key role in FE of pigs.

#### 4.2.2 Hormone-Related Pathways Involved in Feed Efficiency

The brain plays a significant role in regulating feed intake. The brain central nervous system interacts with other organs or tissues such as liver, pancreas and adipose tissue to control appetite and energy balance (Berthoud, 2002). Considering the molecular perspective, signaling molecules of the bilateral gut-brain axis contribute to the regulation of feed intake. In this case, nutrient availability is influenced by the efficiency of enteral absorption processes, including factors such as leptin, ghrelin, neuropeptide Y and cholecystokinin (Richards and Proszkowiec-Weglarz, 2007). Recently, Xu et al. (2020b) revealed that the most significantly enriched pathways were mainly related to hormone secretion, including insulin secretion, GnRH secretion, aldosterone synthesis and secretion, Oxytocin signaling pathway, and pancreatic secretion. Among all significant pathways, insulin secretion and oxytocin signaling pathway are the main pathways that were closely associated with FE. Insulin is a key metabolic hormone that regulates various growth processes through related central actions in the brain (Schwartz et al., 2000). Insulin receptors are expressed in the mesolimbic system of the ventral tegmental area and the arcuate nucleus of the hypothalamus, which respond to insulin to inhibit feed intake and regulate energy homeostasis (Kyriaki, 2003; Davis et al., 2010). Studies by Do et al. (2014a; 2014b) indicated that genes involved in insulin signaling and energy metabolism pathway play key roles in regulating FE-related traits such as RFI and FCR.

#### 4.2.3 Immune-Related Pathways Involved in Feed Efficiency

Maintaining a competent immune system for effective immune response is a nutritionally demanding process that involves trade-offs with other processes such as growth, reproduction and thermoregulation (Lochmiller and Deerenberg, 2000). Exposure to pathogenic challenges creates a situation in which pigs need nutrients for functions that enable defence, including innate immune response, replenishment of damaged or lost tissue, and acquired immune response (Kyriazakis et al., 2006). Therefore, sustaining an appropriate immune response leads to fewer nutrients available for growth (Patience et al., 2015). In this context, the modification of nutrients toward immune response might have negative impacts on the FE of animals (Paradis et al., 2015). suggested that high feed efficient animals had efficient immune response against inflammation, and thus, there was more energy for growth and muscle deposition. Blood cells constitute the first line of the immune defence system; hence there is expectation for numerous genes associated with immunity in the whole blood transcriptome analysis (Liew et al., 2006). The role of nuclear factor of activated T cells (NFAT) in the regulation of the immune response was identified as a significant pathway in the transcriptome analysis of liver tissue in pigs divergent for FE (Horodyska et al., 2019a). NFAT proteins contribute to innate immune-response regulation in the first line of defence through controlling innate leukocyte response to inflammatory stimuli (Zanoni and Granucci, 2012). In the RFI context, transcriptomic studies reported variable and conflicting results for immune-related genes and pathways. Some studies presented only up-regulation of immune-related genes in more efficient pigs (Horodyska et al., 2018). However, in general, studies reported reduced expression of genes associated with immune functions in more efficient pigs (Jégou et al., 2016; Gondret et al., 2017).

#### 4.2.4 Other Pathways

Small intestine is the vital organ where much of the digestion and absorption of feed takes place. The ability of small intestine for digestion and absorption is tightly related to FE traits. Recently, Wu et al. (2020) examined differentially expressed proteins in the small intestine of high-FE against low-FE commercial pigs. Their results showed that regulation of actin cytoskeleton, focal adhesion, adherens junction, and tight junction pathways were the main biological pathways associated with small intestinal structures. Several studies have shown that microvilli, focal adhesions, and intestinal mucosa are key factors that regulate the absorption of nutrients in the small intestine of pigs (Athman et al., 2002; Dokladny et al., 2016). Bruewer et al. (2004) reported that tight junctions and adherens junctions are the main components of apical complex, regulating epithelial paracellular permeability. Tight junctions serve as a selective permeability barrier and regulate the permeability of the intestinal mucosa, which in turn control the entry of small molecules and ions into the body (Andersson, 2009; Dokladny et al., 2016).

## 5 Genetic Improvements in Feed Efficiency

### 5.1 Implementation of Genomic Selection

Although FE has already been improved in the pig industry using traditional selection methods, genomic selection provides further genetic improvements since it only needs phenotypic records for the reference population (Samorè and Fontanesi, 2016). Genetic improvement depends on existing genetic variation, selection intensity, accuracy of the breeding values, and generation interval. The most significant impact of genomic selection on the dairy cattle industry relies on reducing the generation intervals; however, for pigs the impact has been to increase the accuracy of selection for economically important traits, like FE (Samorè and Fontanesi, 2016). The accurate estimation of genomic estimated breeding values (GEBVs) depends on several factors, including the heritability of the trait, the number of included phenotypes, the extent of the linkage disequilibrium between SNP and quantitative trait loci, the size of the training population, and the relationship between the training and reference population (Daetwyler et al., 2008; Pszczola et al., 2012). Table 1 summarizes the results of genomic selection studies that have been conducted on FE traits in pigs.

**TABLE 1:** Literature estimates of genomic estimated breeding values (GEBVs) for feed efficiency trait in pigs.

Traits	Methods	Breeds	Number of samples	Accuracy	Bias	References
**RFI**	ssGBLUP	French Large White pigs	References data set = HRFI line: 398 LRFI line: 399 Validation data set = HRFI line: 400 LRFI line: 433	HRFI line: 0.63LRFI line: 0.22	HRFI line: 0.98 LRFI line: 0.72	Aliakbari et al. (2020)
**RFI**	Bayes A		References data set = 1,047 Validation data set = 516	0.09	-	Jiao et al. (2014a)
**RFI**	GBLUP, Bayesian LASSO, Bayesian A, B and Cπ	Duroc	References data set = 968Validation data set = 304	GBLUP: 0.51 Bayes A: 0.53 Bayes B: 0.51 Bayes LASSO: 0.50 Bayes Cπ1.1*: 0.52Bayes Cπ10.1: 0.51Bayes Cπ100.1: 0.53	GBLUP: 1.23 Bayes A: 1.08 Bayes B: 1.23 Bayes LASSO: 1.19 Bayes Cπ1.1*: 1.24 Bayes Cπ10.1: 1.23 Bayes Cπ100.1: 1.18	Do et al. (2015)
**FCR**	ssGBLUP	French Large White pigs	References data set = HRFI line: 398 LRFI line: 399 Validation data set = HRFI line: 400 LRFI line: 433	HRFI line: 0.41LRFI line: 0.28	HRFI line: 0.74LRFI line: 0.61	Aliakbari et al. (2020)
**FCR**	Bayes A		References data set = 1,047 Validation data set = 516	0.11	-	Jiao et al. (2014a)
**FCR**	GBLUP, Original single-step, Adjusted single-step	Duroc	References data set = 921 Validation data set = 553	Univariate= GBLUP: 0.21Original single-step: 0.22 Adjusted single-step: 0.23Bivariate= GBLUP: 0.18Original single-step: 0.22 Adjusted single-step: 0.22	Univariate= GBLUP: 0.57 Original single-step: 0.89 Adjusted single-step: 0.92 Bivariate= GBLUP: 0.53 Original single-step: 0.98 Adjusted single-step: 0.94	Christensen et al. (2012)
**FCR**	GBLUP, Bayesian LASSO, MIXTURE	Duroc	References data set = 1,375 Validation data set = 536	EBV= GBLUP: 0.40 Bayesian Lasso: 0.40MIXTURE: 0.38Deregressed EBV= GBLUP: 0.44Bayesian LASSO: 0.44MIXTURE: 0.45		Ostersen et al. (2011)

* Parameter with a uniform (0, 1) prior distribution.

Christensen et al. (2012) reported that including genomic information improves prediction for FE traits in Danish Duroc pigs compared with the pedigree-based method. The authors indicated that the accuracy of GEBV was the highest for single-step method (commonly known as ssGBLUP), which is a developed model that can simultaneously implement both pedigree and genomic information. Several statistical methods have been developed to increase the accuracy of GEBV achieved in genomic prediction (Miar et al., 2015; VanRaden, 2020). In this context, studies tested various statistical genomic selection methods for different FE components in pigs (Ostersen et al., 2011; Do et al., 2015). Do et al. (2015) performed a genomic selection study to examine the performance of different genomic prediction methods such as GBLUP, Bayesian LASSO, Bayes A, Bayes B and Bayes Cπ for FE traits in Danish Duroc pigs and reported that the accuracies ranged from 0.508 to 0.531, 0.506 to 0.532, and 0.276 to 0.357 for DFI, RFI and ADG, respectively. Their results indicated that the choice of statistical method had low impact on the accuracy of prediction for FE traits in pigs. Similarly, Ostersen et al. (2011) reported equal performances for three different statistical methods including GBLUP, Bayesian LASSO and MIXTURE. Using purebred pig data with their high relationship with genotyped animals and the traits with strong selection background are the possible reasons for the small differences between the statistical methods. Zhang et al. (2018) investigated the possible increase of genetic gain for different FE components in pigs and indicated that the genetic architecture of FE traits might be the reason for the accuracies differences.

### 5.2 Future Perspectives and Opportunities

#### 5.2.1 Phenotypic Measures and Genetic Analyses

As discussed earlier, several internal and external factors such as body composition, physical activity, maintenance requirements, digestibility, immune response, and measurement errors might affect FE in pigs (Knap and Wang, 2012). Therefore, to develop global metrics for FE traits, it is essential to consider all relevant factors. To date, several ratio and residual traits have been developed as FE measures. Although ratio traits have some advantages, such as the ease of calculation and interpretation, their main drawback is the strong correlations between ratio traits and their component traits (Berry and Crowley 2013). On the other hand, FE can be measured independently of production level using residual measurements like RFI, which are based on the mathematical model considering energy requirements for body maintenance and production over a specific production time period. As the model implies in RFI, the feed intake and production correlation are assumed to be constant at all production and feed intake levels. However, the true biological efficiency depends on the degree of production and feed intake, which means that the correlation of maintenance requirement, production and feed intake varies over the growth period (Van Der Werf, 2004). In this context, it is suggested to apply a random regression model as it considers the variation within animals between different growth period stages (Veerkamp and Thompson, 1999).

Feed intake, and accordingly FE traits, are considered difficult and expensive to measure traits in all pig production farms. Over recent decades, many feeding strategies have been introduced by breeding companies to facilitate the measuring feeding process in a precise way to achieve accurate measurement of individual FE on a large scale that can increase the genetic gain and productivity. Nowadays, a vast amount of data can be extracted from the state-of-the-art technologies such as sensors for precision feeding systems, machine vision sensors, infrared thermal imaging sensors, microphones, and radio frequency identification (RFID) tags. Introducing new traits for selection of FE can significantly improve the accuracy of prediction in pigs. Martinsen et al. (2015) introduced new FE traits like fat efficiency and lean meat efficiency. Their results indicated that these traits help breeders select animals with high genetic potential for efficient deposition of lean meat at low feed costs. Furthermore, measuring the components of FE such as net FE (digestibility, net energy, heat production, and methane energy output), activity and behaviour (feeding per day, total time spent eating per day, feed intake, and time spent eating per visit), and robustness, might help breeders select more efficient individuals in diverse breeding conditions. It was shown that there is positive phenotypic and genetic correation between feeding behaviour and FE traits, indicating their possible role on selection of feed-efficient individuals (Von Felde et al., 1996; Lu et al., 2017). A deep understanding of the feeding behaviour can help breeders to improve feeding strategies, and thus, increase the productivity (Andretta et al., 2016).

#### 5.2.2 Beyond Genomics

Although significant progress has been achieved in understanding the complexities of genetic control of FE traits, the breeders are always seeking ways to improve FE in their breeding programs to obtain greater genetic progress. One practical way to enhance genetic gain is to maintain genetic variation. Gene editing technology, which generates progeny with selected mutations, can add variation to the population (Wang et al., 2015b). Modification of genes might allow breeders to confer the desired phenotypes to pigs to improve production traits or disease resistance. Recently, Zhu et al. (2020) successfully knocked out the myostatin gene (*MSTN*) in Chinese Bama pigs, which substantially accelerated the growth rate and muscle growth. It is acknowledged that genome editing tools can protect pigs from porcine reproductive and respiratory syndrome (PRRS), which is a viral disease affecting domestic pigs (Whitworth et al., 2016). Several studies have shown that modification of CD163 gene inhibits PRRS, and accordingly have positive impacts on growth rate in pigs (Chen et al., 2019; Guo et al., 2019). In a similar fashion, working on the modification of the pig genome, researchers can improve the FE of pigs in the future.

However, the introduction of gene-editing technology will need to meet the global concerns of using this technology in food products and determine well-designed breeding programs offered by top breeding companies. In recent years, numerous “omics” technologies such as proteomics, transcriptomics, metabolomics, epigenomics, and metagenomics have generated valuable data in the research of FE in pigs. For instances, transcriptomic studies have reported many non-coding RNAs involving in regulation of feed efficiency in pigs (Hu G et al., 2022). Over time, integrating such technologies can give us more accurate selection of animals with better FE. The integration, joint modeling, and analyses of different “omics” data through system genetics would increase the power of identifying causal genes, regulatory networks and pathways that might lead to improve economically important traits like FE. Ultimately, the information derived from the integration such as biomarkers and gene networks or causal genes and variants can be incorporated into genomic selection programs to achieve higher accuracy and genetic gain in the pig industry.
