# Supplementary material for: Application of Genetic, Genomic and Biological Pathways in Improvement of Swine Feed Efficiency
Source: Front Genet. 2022 Jun 9;13:903733. doi: 10.3389/fgene.2022.903733 (PMC9220306; doi:10.3389/fgene.2022.903733)
Supplement: Supplementary file 1 [file DataSheet1.docx]

**Table S1.** Literature for estimated heritability of feed efficiency indicators and related traits.

| Traits | | Reference | Breed | h^2^±SE^1^ |  |
| --- | --- | --- | --- | --- | --- |
| Feed conversion ratio | | (Bouquet et al., 2022) | Large White | (0.34±0.04)-(0.42±0.05) |  |
|  | | (Hong et al., 2021) | Duroc | 0.49 |  |
|  | | (Homma et al., 2021) | Landrace-Duroc-Large White | (0.22±0.06)-(0.39±0.06) |  |
|  | | (Santiago et al., 2021) | Yorkshire-Landrace-Duroc | (0.22±0.16)-(0.39±0.18) |  |
|  | | (Déru et al., 2020) | Large White | (0.27±0.11)-(0.47±0.08) |  |
|  | | (Herrera-Cáceres et al., 2019) | Duroc | 0.21±0.09 |  |
|  | | (Godinho, et al., 2018) | Duroc-Large White | (0.15±0.02)-(0.17±0.01) |  |
|  | | (Desire et al., 2015) | Yorkshire-Landrace | 0.26±0.05 |  |
|  | | (Miar et al., 2014) | Duroc-Landrace-Large White | 0.20±0.06 |  |
|  | | (Jiao et al., 2014a) | Duroc | 0.32±0.09 |  |
|  | | (Shirali et al., 2014) | Pietrain-Leicoma | 0.26±0.20 |  |
|  | | (Do et al., 2013a) | Duroc-Landrace-Yorkshire | (0.30±0.04)-(0.32±0.05) |  |
|  | | (Saintilan et al., 2013) | Landrace-Large White-Piétrain | (0.30±0.03)-(0.40±0.06) |  |
|  | | (Gjerlaug-Enger et al., 2012) | Landrace-Duroc | (0.29±0.04)-(0.42±0.05) |  |
|  | | (Saintilan et al., 2011) | Piétrain | 0.47±0.07 |  |
|  | | (Bunter et al., 2010) | Yorkshire | 0.26±0.07 |  |
|  | | (Habier et al., 2009) | Piétrain | (0.13±0.02)-(0.23±0.01) |  |
|  | | (Cai et al., 2008) | Yorkshire | 0.17±0.07 |  |
|  | | (Gilbert et al., 2007) | Large White | (0.31±0.05)-(0.45±0.07) |  |
|  | | (Schulze et al., 2003) | Landrace-Large White | 0.28±0.04 |  |
|  | | (Hermesch et al., 2000) | Landrace-Large White | 0.15±0.04 |  |
|  | | (Johnson et al., 1999) | Large White | 0.26 |  |
|  | | (Labroue et al., 1997) | Landrace-Large White | (0.19±0.02)-(0.20±0.03) |  |
|  | | (Von Felde et al., 1996) | Landrace-Large White | 0.19±0.06 |  |
|  | |  |  |  |  |
| Residual feed intake | | (Hong et al., 2021) | Duroc | 0.51 |  |
|  | | (Homma et al., 2021) | Landrace-Duroc- Large White | (0.22±0.06)-(0.50±0.05) |  |
|  | | (Santiago et al., 2021) | Yorkshire-Landrace-Duroc | (0.19±0.10)-(0.34±0.16) |  |
|  | | (Willson et al., 2020) | Duroc | 0.28±0.07 |  |
|  | | (Déru et al., 2020) | Large White | (0.34±0.05)-(0.41±0.13) |  |
|  | | (Nascimento et al., 2019) | Yorkshire | 0.30±0.09 |  |
|  | | (Godinho et al., 2018) | Duroc-Large White | (0.15±0.01)-(0.19±0.02) |  |
|  | | (Lu et al., 2017) | Duroc | (0.53±0.03)-(0.55±0.03) |  |
|  | | (Hsu et al., 2015) | Yorkshire | 0.28±0.05 |  |
|  | | (Jiao et al., 2014a) | Duroc | 0.10±0.05 |  |
|  | | (Do et al., 2013a) | Duroc-Landrace-Yorkshire | (0.34±0.04)-(0.39±0.05) |  |
|  | | (Saintilan et al., 2013) | Landrace-Large White-Piétrain | (0.21±0.03)-(0.33±0.06) |  |
|  | | (Saintilan et al., 2011) | Piétrain | 0.40±0.06 |  |
|  | | (Bunter et al., 2010) | Yorkshire | 0.25±0.06 |  |
|  | | (Hoque et al., 2009) | Duroc | (0.20±0.07)-(0.38±0.05) |  |
|  | | (Cai et al., 2008) | Yorkshire | 0.29±0.07 |  |
|  | | (Hoque et al., 2007) | Duroc | (0.27±0.03)-(0.42±0.13) |  |
|  | | (Gilbert et al., 2007) | Large White | (0.14±0.03)-(0.24±0.03) |  |
|  | | (Nguyen et al., 2005) | Large White | (0.22±0.08)-(0.24±0.08) |  |
|  | | (Johnson et al., 1999) | Large White | (0.10)-(0.17) |  |
|  | | (Von Felde et al., 1996) | Landrace-Large White | 0.18±0.03 |  |
|  | |  |  |  |  |
| Other traits: | |  |  |  |  |
| Average daily gain | | (Bouquet et al., 2022) | Large White | 0.37±0.04 |  |
|  | | (Homma et al., 2021) | Landrace-Duroc- Large White | (0.41±0.07)-(0.55±0.06) |  |
|  | | (Alam et al., 2021) | Duroc-Landrace-Yorkshire | (0.34±0.00)-(0.36±0.01) |  |
|  | | (Déru et al., 2020) | Large White | (0.27±0.11)-(0.40±0.06) |  |
|  | | (Santiago et al., 2021) | Yorkshire-Landrace-Duroc | (0.16±0.10)-(0.38±0.17) |  |
|  | | (Willson et al., 2020) | Duroc | 0.28±0.07 |  |
|  | | (Nascimento et al., 2019) | Yorkshire | 0.41±0.08 |  |
|  | | (Godinho et al., 2018) | Duroc-Large White | (0.23±0.01)-(0.26±0.01) |  |
|  | | (Lu et al., 2017) | Duroc | (0.35±0.03)-(0.47±0.03) |  |
|  | | (Chang et al., 2017) | Duroc-Landrace-Yorkshire | (0.49±0.04)-(0.58±0.02) |  |
|  | | (Desire et al., 2015) | Yorkshire-Landrace | 0.48±0.06 |  |
|  | | (Cabling et al., 2015) | Duroc | 0.67±0.06 |  |
|  | | (Miar et al., 2014) | Duroc-Landrace-Large White | 0.30±0.08 |  |
|  | | (Shirali et al., 2014) | Pietrain-Leicoma | 0.64±0.19 |  |
|  | | (Do et al., 2013a) | Duroc-Landrace-Yorkshire | (0.32±0.04)-(0.54±0.05) |  |
|  | | (Saintilan et al., 2011) | Piétrain | 0.58±0.07 |  |
|  | | (Hoque et al., 2009) | Duroc | 0.48±0.03 |  |
|  | | (Cai et al., 2008) | Yorkshire | 0.42±0.08 |  |
|  | | (Gilbert et al., 2007) | Large White | (0.35±0.06)-(0.37±0.05) |  |
|  | | (Schulze et al., 2003) | Landrace-Large White | 0.36±0.04 |  |
|  | | (Labroue et al., 1997) | Landrace-Large White | (0.31±0.02)-(0.41±0.04) |  |
|  | | (Von Felde et al., 1996) | Landrace-Large White | 0.43±0.04 |  |
|  | |  |  |  |  |
| Daily feed intake | | (Bouquet et al., 2022) | Large White | (0.46±0.05)-(0.56±0.05) |  |
|  | |  |  |  |  |
|  | | (Homma et al., 2021) | Landrace-Duroc-Large White | (0.31±0.07)-(0.57±0.05) |  |
|  | | (Cheng et al., 2021) | Landrace-Large White | 0.35±0.05 |  |
|  | | (Déru et al., 2020) | Large White | (0.36±0.12)-(0.53±0.06) |  |
|  | | (Nascimento et al., 2019) | Yorkshire | 0.50±0.09 |  |
|  | | (Godinho et al., 2018) | Duroc-Large White | (0.23±0.02)-(0.28±0.03) |  |
|  | | (Zhang et al., 2016) | Landrace | 0.34±0.01 |  |
|  | | (Gourdine et al., 2017) | Large White | 0.26±0.08 |  |
|  | | (Lu et al., 2017) | Duroc | 0.18±0.03 |  |
|  | | (Desire et al., 2015) | Yorkshire-Landrace | 0.40±0.06 |  |
|  | | (Jiao et al., 2014a) | Duroc | 0.66±0.11 |  |
|  | | (Do et al., 2013a) | Duroc-Landrace-Yorkshire | (0.41±0.04)-(0.56±0.04) |  |
|  | | (Saintilan et al., 2013) | Landrace-Large White-Piétrain | (0.21±0.06)-(0.48±0.06) |  |
|  | | (Saintilan et al., 2011) | Piétrain | 0.54±0.06 |  |
|  | | (Bunter et al., 2010) | Yorkshire | 0.45±0.08 |  |
|  | | (Hoque et al., 2009) | Duroc | 0.49±0.06 |  |
|  | | (Cai et al., 2008) | Yorkshire | 0.51±0.08 |  |
|  | | (Gilbert et al., 2007) | Large White | (0.23±0.04)-(0.25±0.10) |  |
|  | | (Schulze et al., 2003) | Landrace-Large White | 0.39±0.03 |  |
|  | | (Hermesch et al., 2000) | Landrace-Large White | 0.23±0.04 |  |
|  | | (Johnson et al., 1999) | Large White | 0.22 |  |
|  | | (Von Felde et al., 1996) | Landrace-Large White | (0.16±0.06)-(0.30±0.05) |  |
|  | |  |  |  |  |
|  |  |  |  |  |  |

^1^SE = standard error

**Table S2.** Summary of genome-wide association studies of FE traits based on the pig QTL database and additional recent papers.

| **Chr** | | **Trait** | **QTL/SNP position (Mbp)** | **QTL span (Mbp)** | **Candidate gene** | **Variance** | **Reference** |  | |  |
| --- | --- | --- | --- | --- | --- | --- | --- | --- | --- | --- |
|  |  |  |  |  |  |  |  |  |  |  |
| **1** | | **RFI** | 18.87 | - | *FBXO33* | 4.37 | (Ding et al., 2018) |  | |  |
|  | |  | 23.10 | - | *NMBR* | - | (Bai et al., 2017) |  | |  |
|  | |  | 304.66 | - | *PRDM12, EXOSC2* | 0.06 | (Sato et al., 2016) |  | |  |
|  | |  | 30.73 | - | *PEX7* | 0.19 | (Do et al., 2014a) |  | |  |
|  | |  | 60.86 | - | *NT5E* | 0.15 | (Do et al., 2014a) |  | |  |
|  | |  | 64.09 | - | *GABRR2* | - | (Do et al., 2013b) |  | |  |
|  | |  | 9.32 | - | *MAS1* | - | (Do et al., 2014b) |  | |  |
|  | | **FCR** | - | 176.01-181.36 | *MC4R, RNF152, PMAIP1* | - | (Silva et al., 2019) |  | |  |
|  | | **ADFI** | 203.36 | - | *ACER2* | - | (Fu et al., 2020) |  | |  |
|  | |  | 178.6 | - | *MC4R* | 0.86 | (Reyer et al., 2017) |  | |  |
|  | |  | 177 | - | *MC4R* | 0.77 | (Onteru et al., 2013) |  | |  |
|  | | **ADG** | - | 191.9-193.8 | *AGBL1, ESR2* | 1.40 | (Bergamaschi et al., 2020) |  | |  |
|  | |  | 306.86 | - | *GTF3C5* | - | (JianPing et al., 2018) |  | |  |
|  | |  | - | 176.19-177.76 | *MC4R, PHLPP1* | - | (Howard et al., 2015) |  | |  |
|  | |  | - | 166-170 | *SOCS6, DOK6* | - | (Jiao et al., 2014b) |  | |  |
|  | |  | 184.40 | - | *CORO2B* | - | (Fontanesi et al., 2014) |  | |  |
|  | |  | 177 | - | *MC4R* | 2.4 | (Onteru et al., 2013) |  | |  |
|  | |  |  |  |  |  |  |  | |  |
| **2** | | **RFI** | 14.61 | - | *KCTD16* | - | (Bai et al., 2017) |  | |  |
|  | |  | - | 111.01-111.94 | *SLCO4C1* | 0.33 | (Onteru et al., 2013) |  | |  |
|  | | **FCR** | - | 144.71-144.88 | *SPOCK1* | - | (Sahana et al., 2013) |  | |  |
|  | | **ADG** | 147.11 | - | *PRELID2* | - | (Fu et al., 2020) |  | |  |
|  | |  | - | 130.75–132.35 | *SLC27A6* | 1.91 | (Ruan et al., 2021) |  | |  |
|  | |  | - | 149.94–151.54 | *ADRB2* | 1.49 | (Ruan et al., 2021) |  | |  |
| **3** | | **RFI** | 68.29 | - | *HK2* | - | (Fu et al., 2020) |  | |  |
|  | | **FCR** | 98.84 | - | *MSH6* |  | (Ding et al., 2018) |  | |  |
|  | | **ADFI** | 68.29 | - | *HK2* | - | (Fu et al., 2020) |  | |  |
|  | | **ADG** | 46.52 | - | *STARD7* | - | (Tang et al., 2019) |  | |  |
|  | |  |  |  |  |  |  |  | |  |
| **4** | | **FCR** | 87.02 | - | *MCM4* | - | (Horodyska et al., 2017) |  | |  |
|  | |  | 112.04 | - | *TBX15* | 2.82 | (Wang et al., 2015a) |  | |  |
|  | |  | 105.56 | - | *IVL* | 2.74 | (Wang et al., 2015a) |  | |  |
|  | | **ADFI** | 12.17 | - | *MYC* | - | (Ding et al., 2017) |  | |  |
|  | | **ADG** | - | 4.38–5.98 | *FAM135B* | 2.56 | (Ruan et al., 2021) |  | |  |
|  | |  | 70.46 | - | *EYA1* | - | (Ji et al., 2019) |  | |  |
|  | |  | 114.15 | - | *IGSF3* | - | (Fontanesi et al., 2014) |  | |  |
|  | |  |  |  |  |  |  |  | |  |
| **5** | | **FCR** | 33.89 | - | *CCT2* | - | (Miao et al., 2021) |  | |  |
|  | |  | 36.72 | - | *MYRFL* | - | (Miao et al., 2021) |  | |  |
|  | |  | 36.35 | - | *FRS2* | - | (Miao et al., 2021) |  | |  |
|  | |  | 39.13 | - | *TRHDE* | - | (Miao et al., 2021) |  | |  |
|  | |  | 47.78 | - | *FAR2* | - | (Miao et al., 2021) |  | |  |
|  | |  | - | 14.21-14.29 | *NUAK1* | - | (Sahana et al., 2013) |  | |  |
|  | | **ADG** | 33.59 | - | *HMGA2* | - | (Quan et al., 2018) |  | |  |
|  | |  | 2.74 | - | *EFCAB6* | - | (Fontanesi et al., 2014) |  | |  |
|  | |  |  |  |  |  |  |  | |  |
| **6** | | **RFI** | - | 27.65-28.13 | *FTO* |  | (Sanchez et al., 2014) |  | |  |
|  | | **FCR** | 78.29 | - | *FGR* | - | (Horodyska et al., 2017) |  | |  |
|  | | **ADFI** | 132.72 | - | *LRRC7* | - | (Ding et al., 2017) |  | |  |
|  | | **ADG** | 114.63 | - | *TTR* | - | (Tang et al., 2019) |  | |  |
|  | |  |  |  |  |  |  |  | |  |
| **7** | | **RFI** | 18.37 | - | *PRL* | 2.37 | (Ding et al., 2018) |  | |  |
|  | |  | 97.88 |  | *ZFYVE26* | - | (Do et al., 2014b) |  | |  |
|  | | **FCR** | 124 | - | *GLRX5* | 0.52 | (Reyer et al., 2017) |  | |  |
|  | |  | 34.80 | - | *GRM4* | - | (Guo et al., 2015) |  | |  |
|  | | **ADFI** | 19.77 | - | *NRSN1* | 1.75 | (Ding et al., 2018) |  | |  |
|  | |  | 123.10 | - | *SERPINA3* | - | (Ding et al., 2017) |  | |  |
|  | | **ADG** | - | 139.8-140.7 | *TMEM266, ETFA, ISL2, SCAPER* | 1.32 | (Bergamaschi et al., 2020) |  | |  |
|  | |  | 30.99 | - | *ANKSIA* | 0.13 | (Gong et al., 2019) |  | |  |
|  | |  |  |  |  |  |  |  | |  |
| **8** | | **RFI** | 87.01 | - | *TTC29* | - | (Do et al., 2014b) |  | |  |
|  | | **FCR** | - | 83.61-83.72 | *DCLK2* | - | (Sahana et al., 2013) |  | |  |
|  | | **ADG** | 112.52 | - | *SYNPO2* | - | (Fontanesi et al., 2014) |  | |  |
|  | |  |  |  |  |  |  |  | |  |
|  | |  |  |  |  |  |  |  | |  |
| **9** | | **RFI** | 142.52 | - | *PROX1* | - | (Do et al., 2014b) |  | |  |
|  | | **FCR** | - | 61.47-61.53 | *ETS1* | - | (Sahana et al., 2013) |  | |  |
|  | | **ADG** | 75.48 | - | *C7orf62* | - | Meng et al., 2017 |  | |  |
|  | |  |  |  |  |  |  |  | |  |
| **10** | | **RFI** | 64.56 | 129.12-129.12 | *PRKCQ* | - | (Bai et al., 2017) |  | |  |
|  | | **FCR** | - | 52.58-52.73 | *FRMD4A* | - | (Sahana et al., 2013) |  | |  |
|  | | **ADG** | 20.77 | - | *CAPN2* | - | (Tang et al., 2019) |  | |  |
|  | |  |  |  |  |  |  |  | |  |
| **11** | | **RFI** | 53.79 | - | *PDX1* | - | (Fu et al., 2020) |  | |  |
|  | |  |  |  |  |  |  |  | |  |
|  | |  |  |  |  |  |  |  | |  |
| **12** | | **RFI** | 52.54 | - | *ASGR1* | - | (Bai et al., 2017) |  | |  |
|  | | **FCR** | 18.34 | - | *MAP3K14* | - | (Ding et al., 2017) |  | |  |
|  | | **ADFI** | 14.83 | - | *TEX2, ICAM2* | - | (Fu et al., 2020) |  | |  |
|  | | **ADG** | - | 1.87–4.22 | *GAA* | - | (Silva et al., 2019) |  | |  |
|  | |  |  |  |  |  |  |  | |  |
| **13** | | **RFI** | 194.87 | 194.9-194.9 | *TIAM1* | - | (Bai et al., 2017) |  | |  |
|  | |  | 21.36 | - | *DSCAM* | 0.13 | Do et al., 2014a |  | |  |
|  | |  | 210.53 | - | *HLSC* | 0.13 | Do et al., 2014a |  | |  |
|  | | **FCR** | 3.65 | - | *PLCL2* | - | (Fu et al., 2020) |  | |  |
|  | | **ADG** | 34.88 | - | *TNNC1* | - | (Tang et al., 2019) |  |  | |
|  | |  |  |  |  |  |  |  |  | |
|  | |  |  |  |  |  |  |  |  | |
| **14** | | **RFI** | 140.72 | - | *GRK5* |  | Do et al., 2014b |  |  | |
|  | |  | 59 | - | *GNG4* | 1.16 | (Onteru et al., 2013) |  |  | |
|  | | **FCR** | 40.5 | - | *PLA2G1B, SIRT4* | - | (Fu et al., 2020) |  |  | |
|  | | **ADG** | 10.79 | - | *A1CF* | - | (Fontanesi et al., 2014) |  |  | |
|  | |  |  |  |  |  |  |  |  | |
| **15** | | **RFI** | 1.77 | - | *RND3* | - | (Bai et al., 2017) |  |  | |
|  | |  | 89.62 | - | *GPR155* |  | (Do et al., 2014b) |  |  | |
|  | | **FCR** | 146.4 | - | *DIS3L2* | - | (Horodyska et al., 2017) |  |  | |
|  | |  | 123.67 | - | *MAP2* | 2.74 | (Wang et al., 2015a) |  |  | |
|  | | **ADFI** |  | 131.44-132.17 | *IGFBP5* | - | (Howard et al., 2015) |  |  | |
|  | | **ADG** | 35.22 | - | *GLI2* | - | (Meng et al., 2017) |  |  | |
|  | |  |  |  |  |  |  |  |  | |
| **16** | | **ADG** | - | 18.8-18.9 | *UBE2H, NRF1, SMKR1, STRIP2, AHCYL2* | 1.45 | (Bergamaschi et al., 2020) |  |  | |
|  | |  | 22.72 | - | *NIPBL* | - | (Tang et al., 2019) |  |  | |
|  | |  |  |  |  |  |  |  |  | |
| **17** | | **RFI** | 45.53 | - | *SOGA1* | - | (Do et al., 2014b) |  |  | |
|  | | **FCR** | 20.14 | - | *PLCB1* | 2.66 | (Ding et al., 2018) |  |  | |
|  | | **ADG** | 17.55 | - | *BMP2* | 1.88 | (Lee et al., 2019) |  |  | |
|  | |  | 20 | - | *PLCB1* | - | (Meng et al., 2017) |  |  | |
|  | |  |  |  |  |  |  |  |  | |
| **18** | | **FCR** | 23.53 | - | *SPAM1, HYAL4* |  | (Fu et al., 2020) |  |  | |
|  | | **ADG** | 45.40 | - | *GHRHR* |  | (Fontanesi et al., 2014) |  |  | |
|  | |  |  |  |  |  |  |  |  | |
